# Roles of Macrophages and Exosomes in Liver Diseases

**DOI:** 10.3389/fmed.2020.583691

**Published:** 2020-09-24

**Authors:** Mengyi Shen, Yi Shen, Xiaoli Fan, Ruoting Men, Tinghong Ye, Li Yang

**Affiliations:** ^1^Department of Gastroenterology and Hepatology, Sichuan University-University of Oxford Huaxi Joint Centre for Gastrointestinal Cancer, Frontiers Science Center for Disease-Related Molecular Network, West China Hospital, Sichuan University, Chengdu, China; ^2^Laboratory of Liver Surgery, State Key Laboratory of Biotherapy/Collaborative Innovation Center for Biotherapy, West China Hospital, Sichuan University, Chengdu, China

**Keywords:** macrophages, exosomes, hepatitis virus, alcoholic liver disease, non-alcoholic fatty liver disease, acute liver failure, hepatocellular carcinoma

## Abstract

Exosomes are small discoid extracellular vesicles (EVs) originating from endosomes that are 30–150 nm in diameter and have a double lipid layer. They participate in the immune response, cell migration, cell differentiation, and tumor invasion and mediate intercellular communication, regulating the biological activity of receptor cells through the proteins, nucleic acids, and lipids that they carry. Exosomes also play vital roles in the diagnosis and treatment of liver diseases. Macrophages, which show unique phenotypes and functions in complex microenvironments, can be divided into M1 and M2 subtypes. M1 macrophages function in immune surveillance, and M2 macrophages downregulate the immune response. Recent studies have shown that macrophages are involved in non-alcoholic fatty liver disease, liver fibrosis, and hepatocellular carcinoma. Moreover, several studies have demonstrated that liver diseases are associated with exosomes derived from or transferred to macrophages. This review focuses on the participation of macrophages and exosomes in liver diseases.

## Introduction

Exosomes are small discoid extracellular vesicles (EVs) originating from endosomes that are 30–150 nm in diameter and have a double lipid layer (1). The exosome formation process involves invagination of the cell membrane to form an endosome, which then develops into a multivesicular body (MVB) that subsequently fuses with the cell membrane, releases the particles outside of the cell, and forms the exosome (2). A variety of cells can secrete exosomes under normal and pathological conditions (3). In addition, exosomes are also widely found in bodily fluids, including blood, saliva, urine, ascites, and cerebrospinal fluid (4, 5). The function of an exosome depends on the type of cell from which it originates. In general, exosomes can participate in processes such as immune response, cell migration, cell differentiation, and tumor invasion (6). Exosomes mediate intercellular communication, regulating the biological activity of receptor cells through the proteins, nucleic acids, and lipids they carry (7, 8). Exosomes also play a vital role in the diagnosis and treatment of liver diseases (9).

Macrophages are a heterogeneous population of cells that exhibit a unique phenotype and function in the complex microenvironment *in vivo*. According to differences in their activation state and function, macrophages can be divided into classically activated macrophages (CAMs or M1) and alternatively activated macrophages (AAMs or M2). M1 macrophages participate in the immune response and in immune surveillance by presenting antigens and secreting pro-inflammatory cytokines such as IL-6 and tumor necrosis factor-α (TNF-α). M2 macrophages have a weak antigen presentation ability and play an important role in immune regulation by downregulating the immune response *via* the secretion of the inhibitory cytokines IL-10, transforming growth factor-β (TGF-β) and mannose receptor (Mrc) (10–12). It has been suggested that macrophages have a series of continuous functional states, and M1 and M2 macrophages are the two extremes of this continuous state (13). Moreover, recent studies have found that macrophages are involved in non-alcoholic fatty liver disease (NAFLD) (14, 15), liver fibrosis (16), and hepatocellular carcinoma (HCC) (17).

Exosomes secreted by hepatocytes exposed to alcohol can be ingested by macrophages, thereby promoting the secretion of cytokines (18). In cholestatic liver disease, exosomal long non-coding RNA (lncRNA) H19 from bile duct cells promotes the M1 polarization of Kupffer cells and the production of chemokine ligand 2 and interleukin 6 (19). In melatonin-treated HCC cells, exosomes change the immunosuppression status of macrophages (20). In this review, we summarize the effects and interaction of macrophages and exosomes in liver diseases (Table 1).

**Table 1:** Summary of exosome and macrophage participation in liver diseases.

**Disease**	**Exosome component**	**Pathway/mediator**	**Effect**	**References**
HBV	HBV-miR-3	SOCS5/STAT1	M1 polarization and IL-6 secretion	(21)
	HBV-infected hepatocyte exosomes	MyD88, TICAM-1, and MAVS	Resistance to the host's inherent immune response	(22)
HCV	Anti-HCV miRNA-29	TLR3-activated macrophages	Inhibition of the HCV replication	(23)
	Exosome-packaged HCV	TLR7/8	Monocytes tend to differentiate into macrophages	(24)
ALD	miR-155	Hsp90	Increase in inflammatory macrophages	(25)
	miR-27A	CD206 on monocytes	M2 polarization	(26)
	CD40L (TNFSF5)	Caspase-3	M1 polarization	(27)
	miR-122	HO-1	Reprogramming ability to make monocytes sensitive to LPS	(18)
	Mitochondrial double-stranded RNA	TLR3 in Kupffer cells	Increase in IL-1β and IL−17A levels	(28)
NAFLD	mi R-192-5p	Rictor/Akt/FoxO1	M1 polarization	(29)
	Hepatocyte-derived EV	DR5/Caspase/ROCK1	Macrophage pro-inflammatory response	(30)
	Lipotoxic EVs	ITGβ1	Promotion of monocyte adhesion and liver inflammation	(31)
	miR122-5p	lysosome	M1 polarization	(32)
ALF	miR-17	TXNIP	Inhibition of inflammatory factor activation in hepatic macrophages	(33)
HCC	lncRNA TUC339	Toll-like receptor signaling and FcγR-mediated phagocytosis	Reduction in pro-inflammatory cytokine production and amelioration of phagocytosis	(34)
	Exo-con	STAT3	Upregulation of PD-L1 expression and cytokine secretion in macrophages	(20)
	miR-23a-3p	PTEN/AKT	Upregulation of PD-L1 expression in macrophages and inhibition of T-cell function	(35)
	miR-142-3p	RAC1	Propofol stimulates the transfer of miR-142-3p from macrophages to HCC cells. MiR-142-3p downregulates RAC1 expression and inhibits HCC cell migration and invasion	(36)

*HBV, Hepatitis B virus; HCV, Hepatitis C virus; ALD, Alcoholic liver disease; NAFLD, Nonalcoholic fatty liver disease; ALF, Acute liver failure; HCC, Hepatocellular carcinoma; Exo-con, Hepatocellular carcinoma-derived exosomes; M1, M1 macrophages; M2, M2 macrophages*.

## Characteristics of Exosomes

Cells release bilayer membranous vesicles called EVs, which can be divided into exosomes, microvesicles (MVs), ectosomes, migrasomes, apoptotic bodies, and oncosomes according to their size and origin (37). Exosomes are the smallest EVs, with a diameter of 30–150 nm. Further, exosomes can be divided into small exosomes (60–80 nm) and large exosomes (90–120 nm). Proteomic analyses have shown that small exosomes carry proteins that are associated with endosomes, MVBs, and phagocytic vesicles, indicating that small exosomes are classical exosomes from the endosomal compartment. In contrast, large exosomes include plasma membrane proteins, cellular connexins, and late endosomal proteins and may be atypical exosomes from plasma membrane germination (38). Medium-sized EVs, 100–1,000 nm in diameter, include MVs, ectosomes, and microparticles (39). Ectosomes depend on the plasma membrane, while exosomes depend on endocytic membranes. These two distinct types of EVs differ in size, composition, and release regulation mechanisms. For ectosomes and exosomes, the goods on the surface and in the lumen differ when EVs are released by different cell types or individual cells in different functional conditions. After release, the two types of EVs move through the extracellular fluid at different times and for different distances (40). Migrasomes, apoptotic bodies, and oncosomes are large EVs (a few thousand nanometers) that have been found to be associated with migration, phagocytosis, and cancer, respectively. Migrasomes are newly identified organelles that depend on migration, leaving long retractable fibers upon cell migration, and vesicles grow atop the tips and intersections of fibers. Eventually, the fibers that connect the vesicles to the main cell body break apart, and the vesicles are released into the extracellular space or absorbed directly by the surrounding cells (41). Apoptotic bodies, small bodies released by programmed cell death, can be formed in two ways: the sprouting and shedding mechanism and the autophagosome mechanism (42). The term “oncosomes” was originally used to describe abnormally large EVs, although it is often used to refer to EVs released by cancer cells (43). Oncosomes derived from prostate cancer cells strongly promote the establishment of a tumor-supporting environment by inducing new interstitial reprogramming (44). In fact, EVs should not be classified into subtypes according to their sizes because their diameters overlap; for example, some MVs, whose size range is very large (100–1,000 nm), can be easily confused with large exosomes (45). At present, the origin is the only basis for distinguishing exosomes from other EVs. Other EVs are formed by the protrusion and shedding of cell membranes, whereas exosomes are derived from intracellular endosomes, which can form MVBs that are then degraded by lysosomes or fused with the cytoplasmic membrane, released and enter the receiving cell through fusion, endocytosis or receptors (46). According to the MISEV2018 guidelines, exploring the biogenesis of EVs remains a challenge without the use of live imaging techniques. Therefore, operational terms are still recommended for the description of EV subtypes according to their size, density, biochemical composition, and cell or organ origin (47).

**Figure 1:**
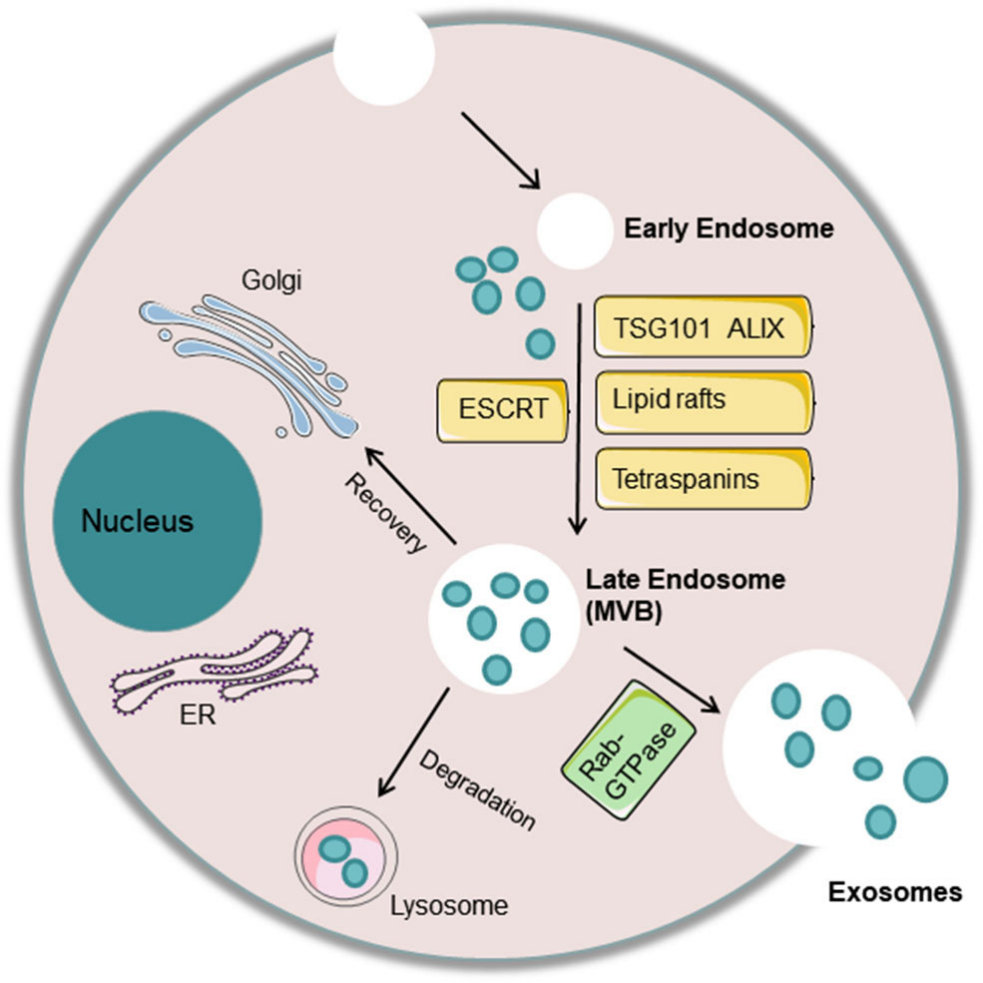
Exosome biogenesis and abscission. The cell membrane invaginates to form an endosome, which then develops into a late endosome or multivesicular body (MVB) with processing of the rough endoplasmic reticulum and Golgi. Some of these MVBs directly fuse with lysosomes and degrade, some are transported to the Golgi for recovery, and some fuse with the cell membrane to release small vesicles outside of the cell and form exosomes. Many molecules play an important role in exosome biogenesis and abscission. First, the endosomal sorting complex required for transport (ESCRT) and other proteins, such as tumor susceptibility gene 101 protein (TSG101) and ALG-2 interacting protein X (ALIX), are involved in cargo sorting into exosomes. In addition, other ESCRT-independent mechanisms, including lipid rafts and tetraspanins CD63 and CD81, are conducive to exosome biogenesis. Finally, the Rab-GTPase family contributes to the intracellular trafficking and fusion of MVBs with the cell membrane to release exosomes.

The exosome formation process involves invagination of the cell membrane to form an endosome, which then develops into MVBs. Some of these MVBs directly fuse with lysosomes and degrade, some are transported to the Golgi for recovery, and some fuse with the cell membrane to release small vesicles outside of the cell and form exosomes. Regarding the mechanisms associated with exosome biogenesis and abscission, many molecules play an important role. First, the endosomal sorting complex required for transport (ESCRT) and other proteins, such as tumor susceptibility gene 101 protein (TSG101) and ALG-2 interacting protein X (ALIX), are involved in cargo sorting into exosomes (3). Apart from ESCRT, other ESCRT-independent mechanisms, including lipid rafts and tetraspanins CD63 and CD81, are conducive to exosome biogenesis (48). Finally, the Rab-GTPase family contributes to the intracellular trafficking and fusion of MVBs with the cell membrane to release exosomes. Some studies clarified that sphingomyelinase, protein kinase D family, and argonaute-2 are involved in the formation of exosomes (49) (Figure 1).

**Figure 2:**
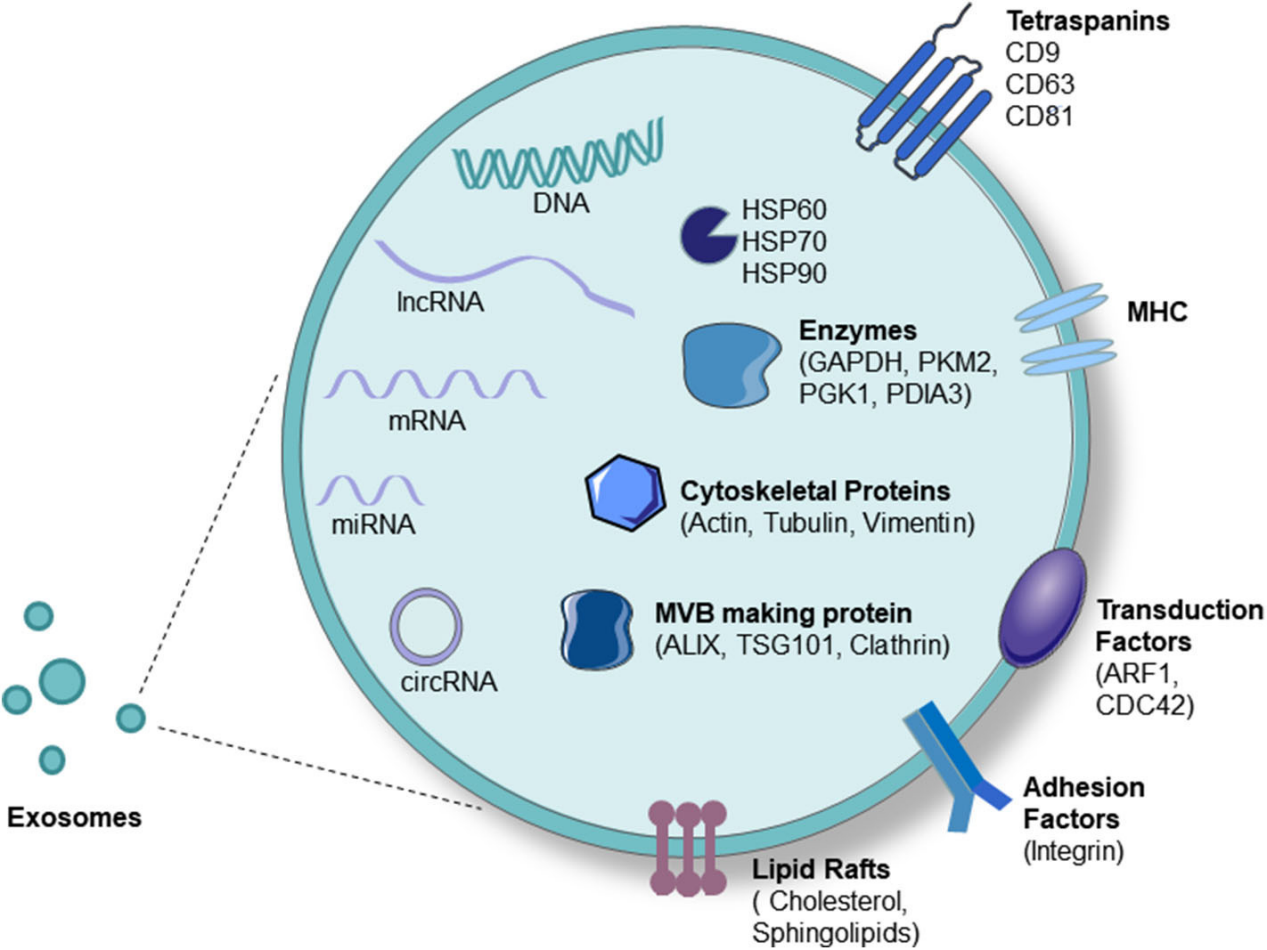
Exosome components. Exosomes carry proteins, nucleic acids, and lipids. Proteins include tetraspanins (CD63, CD81, and CD9), some cell-specific proteins such as MHC-II (antigen-presenting cell source), heat-shock protein family (HSP60, HSP70, HSP90), a variety of metabolic enzymes (GAPDH, PKM2, PGK1, PDIA3, antioxidant proteins), signal transduction factors (ARF1, CDC42), adhesion factors (MFGE8, integrin), cytoskeletal proteins (Actin, Tubulin, Vimentin), and MVB-producing proteins (Alixs, Tsg101, Clathrin). Nucleic acids include DNA, mRNA, microRNA, lncRNA, and circRNA.

Exosomes are composed of nucleic acids (including DNA and RNA), proteins, and lipids. Exosomal RNAs mainly play key roles in the target cell and mainly include mRNAs, microRNAs, lncRNAs, circRNAs, etc. (50). MicroRNAs are now the most widely and deeply studied type of RNA in exosomes, often due to their relationship with the occurrence and development of diseases (51). Exosomal proteins can be divided into membrane proteins and intramembrane proteins. Membrane proteins, including tetraspanins (CD63, CD81, and CD9) and some cell-specific proteins, such as A33 (colon epithelial cell source), MHC-II, and CD86 (antigen-presenting cell sources), participate in exosome transport. Intracellular exosomal proteins include the heat shock protein family (HSP60, HSP70, HSP90, HSPA5, and CCT2), a variety of metabolic enzymes (GAPDH, PKM2, PGK1, PDIA3, antioxidant proteins), ribosomal proteins (RPS3), signal transduction factors (melanoma differentiation-related factors, ARF1, CDC42), adhesion factors (MFGE8, integrin), cytoskeletal proteins, and ubiquitin (52, 53). Lipids are important components of the exosomal membrane, and exosomes contain more specific lipids than parent cells. Several studies have found that the percentages of different lipid categories in cells and exosomes vary among several cell types, such as human B cells and dendritic cells. Specifically, in human B cells, cholesterol, and sphingomyelins have been found to be enriched from cells to exosomes (54) (Figure 2).

Exosomes have several functions. First, they function as a shuttle bus between cells, mediate cell-cell communication and play a role in immunity. Exosomes have been identified as important mediators of intercellular communication through the transfer of encapsulated cargo, such as bioactive lipids, non-coding RNAs, mRNAs, and proteins (55). These bioactive molecules are stable because they are covered with a biofilm capable of avoiding degradation. In addition, due to the characteristics of their surface proteins, exosomes also show relatively high target specificity to receptor cells. All these characteristics make exosomes important mediators of communication between cells, especially between organs. Second, the occurrence and progression of diseases [e.g., tumor metastasis (56–58), cardiovascular disease risk (59, 60), neurological diseases (61, 62)] can be predicted by studying the relationship among the exosome type, number, size, and content. Finally, several recent studies have reported on targeted drugs for exosomes. Exosomes can be used as carriers to deliver drugs to target areas, providing hope for the treatment of many diseases (63, 64).

## Hepatitis Virus

Hepatitis B virus (HBV) infection is characterized by long-term chronic infection accompanied by hepatocyte injury due to the complicated interaction between HBV and the immune system. In addition, according to the World Health Organization, more than 185 million people are infected with hepatitis C virus (HCV) (65). In the process of HCV infection, the interaction between macrophages and hepatocytes is an important part of liver innate immunity.

HBV encodes a microRNA (HBV-miR-3) that inhibits HBV replication by impeding transcription. Type I interferons (IFNs) constitute important immune responses to viral infection and can thus be used to treat some infectious viruses, including HBV and HCV. IFN-I interacts with its receptor to activate the Janus kinase (JAK)/STAT pathway, and STAT1/2 is then phosphorylated and transferred to the nucleus to bind to the IFN-stimulating response element, initiating the transcription of IFN-stimulated genes. SOCS5, an E3 ubiquitin ligase, negatively regulates the mechanism described above; specifically, it inhibits JAK kinase activity by interacting with JAKs through its JAK interaction region. Exosomal HBV-miR-3 promotes macrophage differentiation into the M1 phenotype and IL-6 secretion through the SOCS5/STAT1 pathway (21). Macrophage exosomes rely on T cell immunoglobulin and the hepatitis A virus receptor mucin receptor 1 to enter liver cells and then promote anti-HBV activity induced by IFN-α. In addition, the two main endocytic pathways for viral infection, namely, reticular protein-mediated endocytosis and macrophage phagocytosis, cooperate to allow exosome entry into liver cells and transfer of this activity (66). HBV-infected hepatocyte exosomes carry viral nucleic acids and prompt the expression of NKG2D ligands in macrophages. Compared to normal hepatocytes, HBV-infected hepatocyte exosomes show higher expression levels of immunoregulatory microRNAs, which are transported to macrophages and then restrict IL-12p35 mRNA expression in macrophages, leading to resistance to the host's inherent immune response (22).

Exosomes derived from macrophages play a key role in inhibiting the replication of HCV. TLR3-activated macrophages release exosomes containing anti-HCV microRNA (miRNA)-29 family members (23). Further studies show that interferon-stimulated macrophage-derived EVs inhibit HCV replication and participate in antiviral immune responses, while polyunsaturated fatty acids weaken this process (67). On the other hand, exosomes can also affect macrophages. Concretely, monocytes tend to differentiate into macrophages that show high expression of M2 surface markers and produce pro- and anti-inflammatory cytokines when cocultured with exosome-packaged HCV, which is mediated by TLR7/8 (24).

## Alcoholic Liver Disease

Alcoholic liver disease (ALD) or alcoholic hepatitis (AH) is a liver disease caused by long-term heavy drinking. The effects of alcohol, alcohol metabolites, and gut-derived endotoxins cause liver damage in patients with ALD (68, 69). The initial manifestation is usually fatty liver, which can develop into AH, liver fibrosis, and liver cirrhosis (70). Approximately 3.3 million people die each year from excessive drinking, accounting for almost 5.9% of all global deaths. According to the World Health Organization, Europe has the highest amount of alcohol consumption per adult. In EU countries, 41% of all liver deaths are attributed to alcohol. Since ALD patients have not shown any clinical symptoms or abnormal laboratory indicators in the past, screening should be carried out in high-risk groups (71).

A recent study focused on the potential correlation between autophagy and exosomes since autophagosome and exosome biogenesis involve the same components. The researchers found autophagy damage in ALD and AH mouse models and in the livers of patients with ALD. Moreover, this autophagy occurs at the lysosome level by reducing the expression of lysosome-associated membrane protein 1 (LAMP1) and lysosome-associated membrane protein 2 (LAMP2). The expression of microRNA 155 (miR-155) is increased by alcohol, and its action targets are LAMP1, LAMP2, mechanistic target of rapamycin, and Ras homolog enriched in the brain. In line with this, miR-155 gene-deficient mice exhibited less alcohol-induced autophagic damage and less exosome production than control mice. Downregulation of LAMP1 or LAMP2 increases the number of exosomes released by hepatocytes and macrophages. These results reveal that the increased exosome content induced by alcohol is related to the destruction of autophagy and the impaired function of autophagosomes and lysosomes (25). Another study clarified that atypical exosomes can eliminate lysosomal waste to combat lysosomal dysfunction, thus maintaining dynamic equilibrium (72). In addition, researchers have found that EVs in patients with ALD carry a unique protein cargo and induce macrophage activation by heat shock protein 90 (73). Another study found that alcohol increases the EV (mainly exosomes) production of primary human monocytes and THP-1 monocytes, and monocytes exposed to alcohol communicate with primitive monocytes through EVs. Furthermore, miR-27A in exosomes polarizes primitive monocytes into M2 macrophages (26). Similarly, patients with AH and alcohol-fed mice produced more EVs than normal controls. Exosomal miRNA-192, miRNA-122, and miRNA-30a secreted into the blood could be used as diagnostic biomarkers of ALD (74). In addition, a previous study in mice demonstrated that ethanol promotes the secretion of EVs *via* CYP2E1 and revealed for the first time that the caspase-3 pathway is involved in this process. EVs contain CD40L (TNFSF5) and can activate pro-inflammatory macrophages (27). Liver cells exposed to alcohol secrete exosomes containing increased concentrations of miR-122, which is absorbed by macrophages and makes them sensitive to lipopolysaccharide (LPS), thereby enhancing cytokine secretion (18). In addition, mitochondria have also attracted much attention. Ethanol exposure can activate toll-like receptor 3 in Kupffer cells by hepatic mitochondrial double-stranded RNA (MtdsRNA) through exosomal delivery, resulting in increased IL-1β levels, which promotes the production of IL-17A. MtdsRNA and TLR3 can be used as therapeutic targets for ALD (28, 75). Hence, blocking these pathways may protect against alcohol-induced liver injury.

## Non-Alcoholic Fatty Liver Disease

NAFLD is characterized by the excessive accumulation of liver fat and insulin resistance, which is defined by histological analysis as >5% hepatocyte steatosis or by proton density as a fat content of >5.6%. NAFLD includes two kinds of pathological diagnoses with different prognoses: non-alcoholic fatty liver (NAFL) and non-alcoholic steatohepatitis (NASH). The latter is more severe than the former and includes fibrosis, liver cirrhosis, and HCC (76). High-calorie diets, excessive intake of saturated fats, refined carbohydrates, sugary drinks, and fructose and Western diets are all associated with increased body mass, obesity, and especially NAFLD (77). High-fructose intake increases the risk of NASH and advanced liver fibrosis (78, 79). In addition, it is generally recognized that monocyte-derived macrophages recruited in the liver are involved in the inflammatory response of NASH.

The pathological features of NASH are lipid-induced hepatocyte apoptosis (apoptosis induced by toxic lipid mediators) and infiltration by inflammatory cells, some of which are activated macrophages (80). The latest research indicates that the number and miR-192-5p level of serum exosomes in NASH patients, and NASH model rats are significantly higher than those in their respective control groups. Furthermore, the exosomes released by lipotoxic hepatocytes can be ingested by macrophages, resulting in activation of M1 macrophages and hepatic inflammation by regulating the Rictor/Akt/FoxO1 signaling pathway (29). Another study showed that in a mouse model of NASH, EVs derived from lipotoxic hepatocytes are rich in active integrin β1 (ITGβ1), mediating the adhesion of monocytes to hepatic sinusoidal endothelial cells, which is a necessary step in hepatic inflammation. ITGβ1 inhibition reduces liver injury (31). In addition, it has been reported that exosomes isolated from melatonin-treated adipocytes significantly attenuate liver steatosis induced by a high-fat diet and resistin-mediated ER stress. Further research has shown that melatonin reduces the level of exosomal resistin derived from adipocytes through Bmal1 transcription inhibition and M6A RNA demethylation in adipocytes (81). Several studies have observed that macrophage-derived exosomes contribute to insulin resistance through paracrine or endocrine mechanisms (55, 82, 83). Another study found elevated levels of exosomes derived from natural killer T cells and macrophages among patients with NAFLD or NASH (84). Moreover, lipids have been shown to stimulate death receptor 5, promoting the release of EVs from hepatocytes; subsequently, these EVs activate the inflammatory phenotype in macrophages, which ultimately causes NASH (30). Cholesterol damages the lysosomal function of hepatocytes, leading to the secretion of hepatocyte-derived exosomal miR122-5p, which enters macrophages to promote M1 polarization and the occurrence of inflammation (32). Hepatocytes treated with ezetimibe can inhibit inflammasome formation in macrophages and IL-1 secretion as well as alleviate NASH liver inflammation through exosomes (85).

## Acute Liver Failure

Acute liver failure (ALF), a clinical syndrome characterized by jaundice, ascites, hepatic encephalopathy, and coagulation dysfunction, refers to the extensive necrosis of hepatocytes or severe liver function damage caused by various factors, such as viruses, drugs, and toxins. The treatments for ALF are liver transplantation and artificial liver therapy. However, there are limitations associated with liver transplantation due to a lack of appropriate donor livers and a variety of complications. Additionally, the efficacy of artificial liver therapy is relatively limited (86).

The transplantation of mesenchymal stem cells (MSCs) might become a potential approach for treating liver disease (87). Researchers administered human umbilical cord MSC-derived exosomes (hucMSC-Ex) to mice *via* their tail vein or oral gavage. The hucMSC-Exs exhibited antioxidant functions and antiapoptotic effects and rescued the mice from liver failure induced by CCl4 (88). Another study further explored the role of macrophages in this process. The researchers treated mice with LPS and D-galactosamine (LPS/GalN) and immediately injected adipose MSC (AMSC)-derived exosomes (AMSC-Exos) intravenously. AMSC-Exos colocalized with hepatic macrophages and reduced the secretion of inflammatory factors by inhibiting the activation of inflammatory factors in macrophages. Exosome-encapsulated miR-17 plays an important role in the treatment of ALF by targeting TXNIP and inhibiting the activation of inflammatory factors in hepatic macrophages (33). Exosomes secreted by MSCs may improve the therapeutic efficacy of MSCs by mediating intercellular communication and transporting paracrine factors (89).

## HCC

The incidence of liver cancer is on the rise worldwide, with the number of newly diagnosed cases increasing by 75% between 1990 and 2015 (90). It is predicted that liver cancer will be the sixth most common cancer in the world and the fourth-largest cause of cancer-related death. According to statistics by the International Agency for Research on Cancer, there were approximately 842,080 new cases of liver cancer and 781,631 deaths in 2018. Liver cancer includes HCC (75–85% of cases), intrahepatic cholangiocarcinoma (10–15% of cases) and other rare types (91). Because patients with early HCC exhibit no obvious clinical symptoms, early diagnosis is quite difficult. Currently, screening methods for HCC rely on mainly serum tumor markers and imaging tests. Clinical serological tests include α-fetoprotein (AFP), des-γ-carboxy prothrombin, and the AFP-L3 fraction. Imaging-based diagnostic methods include computed tomography and magnetic resonance imaging. If necessary, pathological examination may be used, but this method is not ideal in early HCC monitoring (92–94). Surgical resection is suggested as the first choice for the treatment of HCC patients with non-cirrhosis. However, those who undergo surgery have a recurrence rate of 70% (95). Therefore, a need exists for improved diagnostic and treatment methods for liver cancer.

Recent studies have shown that tumor-derived exosomes can be absorbed by fibroblasts and macrophages in the tumor microenvironment, change their phenotype, and ultimately promote tumor progression and metastasis (96). Several studies have noted that HCC-derived exosomes can be ingested by macrophages and thereby promote tumor progression. A recent study showed that exosomes derived from HCC contain a large amount of the lncRNA TUC339, which is taken up along with exosomes by macrophages in the tumor microenvironment, reducing the secretion of pro-inflammatory cytokines from these macrophages, increasing the secretion of anti-inflammatory cytokines, and causing the phenotypic conversion of macrophages. These phenotypically transformed macrophages can inhibit immune-mediated tumor cell death and promote tumor immune escape, thus facilitating rapid tumor growth progression (34). The exosomes secreted by hepatoma cells and released by melatonin-induced hepatoma cells can be phagocytosed and ingested by macrophages. The immune response is affected by regulating the expression of PD-L1 and the inflammatory factors IL-6, IL-10, and TNF-α. The melatonin-induced release of exosomes from HCC cells downregulates the expression of PD-L1 in macrophages by downregulating the protein expression of STAT3 (20). Another HCC study showed that endoplasmic reticulum (ER)-stressed HCC cells release exosomes, upregulate PD-L1 expression in macrophages, and then inhibit T cell function through the exosomal miR-23a-PTEN-AKT pathway. These results provide new insights into how tumor cells escape antitumor immunity (35). Hepatoma cells transmit miRNA-21 to hepatic stellate cells and activate the tumor suppressor gene PTEN through exosomes to activate the transition of hepatic stellate cells into cancer-associated fibroblasts (CAFs) via the PDK1/AKT signaling pathway. Activated CAFs further secrete angiogenic cytokines, including vascular endothelial growth factor (VEGF), MMP2, and MMP9, increasing the number of blood vessels and promoting the development of HCC (97). In contrast, the expression of miR-122 in serum or circulating exosomes is lower in HCC patients than in healthy subjects (98, 99). In tumor-bearing mice, propofol inhibits the invasion of HCC cells by stimulating the transfer of microvesicular miR-142-3p from tumor-associated macrophages to HCC cells (36). ER stress induces the release of exosomes from HCC cells, and by regulating the expression of programmed death ligand 1 in macrophages, the mir-23a-PTEN-AKT pathway inhibits T cell function and weakens antitumor immunity (35). Macrophages and exosomes also play an important role in tumor metastasis. Some scholars have found that in pancreatic ductal adenocarcinoma cells, tumor-derived exosomes can recruit bone marrow-derived macrophages to form a preliver metastatic environment and promote tumor metastasis (100).

## Exosome Roles in Prognosis and Treatment

In the process of HCV infection, the interaction between retained macrophages and hepatocytes is an important part of liver innate immunity. Exosomes derived from macrophages play a key role in inhibiting the replication of HCV. Further study shows that TLR3-activated macrophages release exosomes containing anti-HCV miRNA-29 family members (23). Virus entry mechanisms and pathways have also been applied to study the exosome-mediated transfer of antiviral activity between cells. In HBV infection, macrophage-derived exosomes can use hepatitis A virus receptors to enter liver cells. Subsequently, exosomes utilize clathrin-mediated endocytosis and macrophage phagocytosis and then fuse with endosomes to effectively transmit the anti-HBV activity induced by IFN-α (66). Together, these studies suggest that exosomes have great potential as delivery vehicles for disease treatment. Exosomes can also be used for prognostic analyses. Circulating EV concentrations and sphingolipid carrier characteristics can be used not only for the diagnosis and differentiation of AH, decompensated alcoholic cirrhosis, and other end-stage liver diseases but also for the prediction of the 90-day survival time (101).

## Conclusions and Perspectives

**Figure 3:**
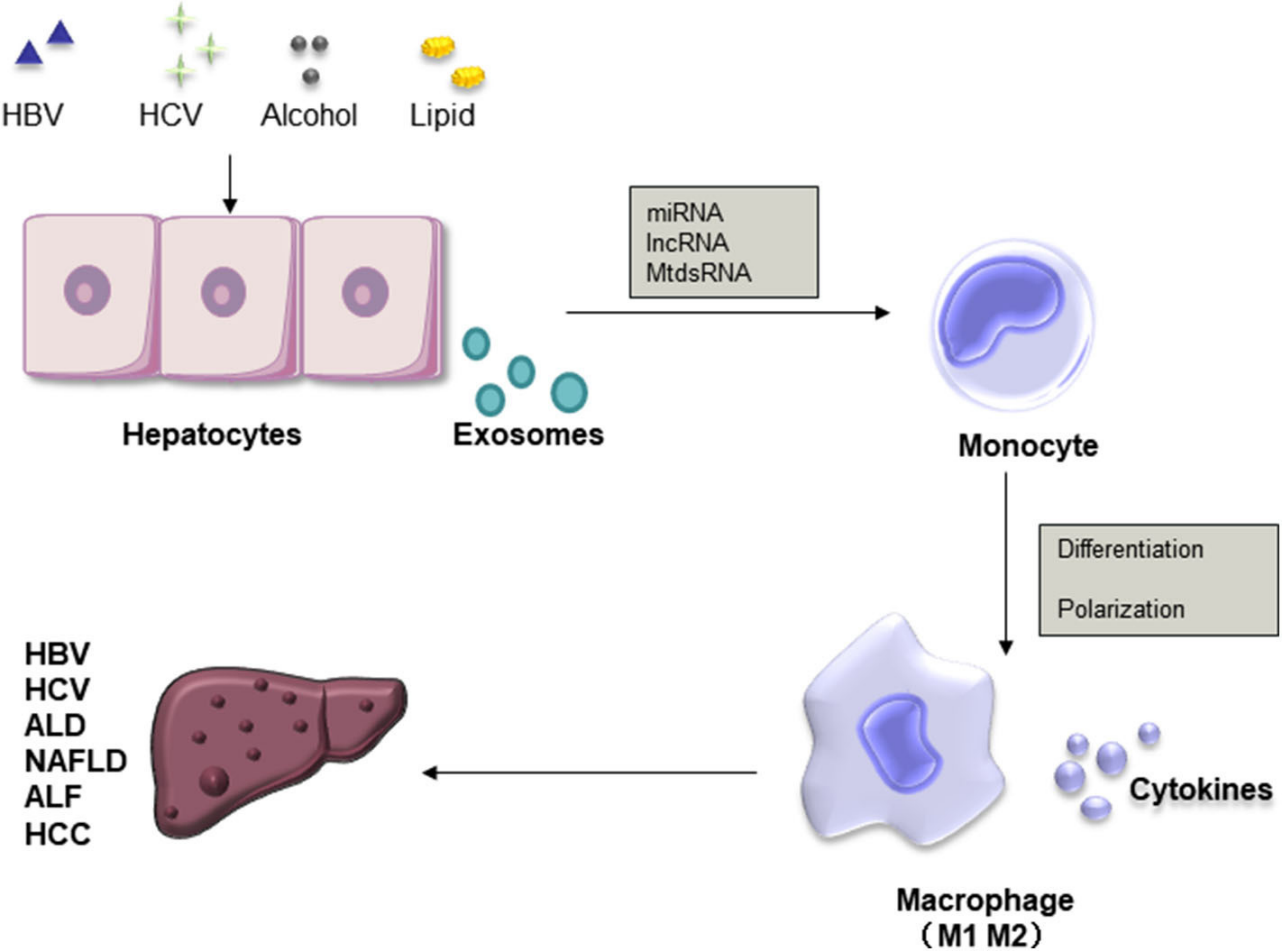
Roles of exosomes and macrophages in liver diseases. Exosomal miRNAs, lncRNAs, and MtdsRNAs released from injured hepatocytes promote the differentiation of macrophages into the M1 or M2 phenotype and the secretion of cytokines, thereby promoting inflammation.

Macrophage activation is an important force driving liver injury. Exosomes are important vesicles that are released by almost all cell types and play an important role in intercellular communication. Increasing evidence indicates that exosomes have outstanding functions, suggesting their potential use for future applications. In all liver diseases, studies on the effects and connections between macrophages and exosomes have concentrated on ALD, NAFLD, and HCC areas and have provided ideas for the non-invasive diagnosis and treatment of these diseases (Table 1). Generally, exosomes from damaged hepatocytes or tumors can promote the activation and differentiation of macrophages, thereby promoting inflammation. On the other hand, macrophage-derived exosomes also play a role in target hepatocytes (Figure 3). Nevertheless, the identification of novel specific biomarkers is required. In addition, it is worth investigating macrophages and exosomes in other liver diseases.

## Author Contributions

MS and YS drafted the manuscript. LY and TY conceived the idea. XF and RM provided critical feedback. All authors read and approved the final version. All authors contributed to the article and approved the submitted version.

## Conflict of Interest

The authors declare that the research was conducted in the absence of any commercial or financial relationships that could be construed as a potential conflict of interest.
